# Effects of workplace-based dietary and/or physical activity interventions for weight management targeting healthcare professionals: a systematic review of randomised controlled trials

**DOI:** 10.1186/s40608-014-0023-3

**Published:** 2014-11-14

**Authors:** Brian T Power, Kirsty Kiezebrink, Julia L Allan, Marion K Campbell

**Affiliations:** Health Services Research Unit, University of Aberdeen, Health Sciences Building, Foresterhill, Aberdeen, AB25 2ZD UK; Health Psychology, Division of Applied Health Sciences, University of Aberdeen, Aberdeen, AB25 2ZD UK

**Keywords:** Workplace, Weight loss, Health Professionals, Systematic review, Diet, Physical activity

## Abstract

**Background:**

The prevalence of overweight and obesity is high amongst healthcare professionals and there is growing interest in delivering weight loss interventions in the workplace. We conducted a systematic review to (i) examine the effectiveness of workplace-based diet and/or physical activity interventions aimed at healthcare professionals and to (ii) identify and describe key components of effective interventions. Seven electronic databases were systematically searched.

**Results:**

Thirteen randomised controlled trials met the inclusion criteria, of which seven had data available for meta-analysis. Where meta-analysis was possible, studies were grouped according to length of follow-up (<12 months and ≥12 months) and behavioural target (diet only, physical activity only or diet and physical activity), with outcome data pooled using a weighted random effects model. Nine studies reported statistically significant (between-group) differences. Four studies reported being informed by a behaviour change theory. Meta-analysis of all trials reporting weight data demonstrated healthcare professionals allocated to dietary and physical activity interventions lost significantly more body weight (−3.95 Kg, [95% CI −4.96 to- 2.95 Kg]) than controls up to 12 months follow up.

**Conclusions:**

Workplace diet and/or physical activity interventions targeting healthcare professionals are limited in number and are heterogeneous. To improve the evidence base, we recommend additional evaluations of theory-based interventions and adequate reporting of intervention content.

**Electronic supplementary material:**

The online version of this article (doi:10.1186/s40608-014-0023-3) contains supplementary material, which is available to authorized users.

## Background

There is increasing recognition that healthcare professionals are role models for the public through their promotion of healthy lifestyle choices [1-4]. Despite this, evidence suggests that 58%-65% of healthcare professionals are overweight or obese [3,5-8], and that many healthcare professionals display poor dietary habits [9] and/or fail to achieve sufficient levels of physical activity [10].

The impact of overweight and obesity in healthcare professionals is significant and wide ranging negatively affecting not only the individual’s own health but also healthcare provider resources, service delivery and patient care [3]. For instance, associations between overweight and obesity and diminished work productivity and occupational injury have been established, with a significantly higher incidence of these issues in the healthcare sector than in other occupations [11,12]. Obesity and concomitant health problems are an important determinant of sickness absence among employees in general [13,14] and healthcare professionals in particular [15]. Amongst healthcare professionals, sickness absence rates are high and reportedly higher than those of employees in the private sector [3,16]. Absenteeism in healthcare professionals may create overload among the healthcare staff that remain, which in turn can adversely affect the delivery of quality care to patients [17]. In addition to the effects on illness and sickness absence outcomes, a systematic review by Zhu et al. [18] suggested that overweight healthcare professionals are less confident delivering weight management advice to patients, perceive more barriers to weight management for their patients, and have fewer positive expectations for patient health outcomes than their healthy weight colleagues.

Considering the high prevalence of overweight and obesity in healthcare professionals and the associated direct and indirect costs, there is a need for effective weight management interventions in this population group. In recent years, interest in using the workplace to deliver such interventions has grown [19]. Several aspects of the workplace are advantageous. For instance, workplaces enable repeated access to a relatively fixed population of workers over extended periods of time [20]. In addition, the workplace allows healthcare providers to demonstrate exemplary practice in supporting the health of their employees while also setting a good example for the patients under their care.

To our knowledge, only one published systematic review has evaluated the possible effects of lifestyle interventions (defined for the purposes of this review as interventions that promote change in lifestyle behaviours such as dietary and physical activity) delivered to healthcare professionals in the workplace [21]. However, the review investigated only interventions targeting nurses. It was also unable to draw definitive conclusions because of the small number of studies identified (n = 3) and the quality of the included studies (none were randomised controlled trials (RCTs) – the acknowledged gold standard design for evaluating healthcare interventions).

As the healthcare workforce is a heterogeneous mix of professions it cannot be assumed that weight problems are confined to nurses. We, therefore, sought to undertake a formal systematic review of published RCTs which evaluated the effectiveness of workplace-based dietary and/or physical activity interventions targeting any healthcare professional group. In addition, our review sought to examine the theoretical underpinning and component parts of the identified interventions to facilitate an understanding of what works and why [22-24].

The formal aims of the systematic review were to: 1) investigate the effectiveness of workplace-based dietary and/or physical activity interventions targeting any healthcare professional groups; 2) identify and describe key components of successful interventions, 3) identify theoretical models of behaviour change involved in effective interventions and 4) investigate whether intervention effectiveness is improved by the extent to which interventions are explicitly developed based on theory.

## Methods

### Study design

The study was a systematic literature review of published studies. We considered ‘published’ studies to be manuscripts that appeared in peer-reviewed journals, dissertations, policy documents or reports. A formal protocol (available on request) was developed prior to undertaking the review and the conduct and reporting of this systematic review adhered to the criteria of the Preferred Reporting Items for Systematic Reviews and Meta-analysis (PRISMA) Statement [25].

### Inclusion criteria

Articles were eligible for inclusion if they described a randomised controlled trial (RCT) comparing a workplace-based diet and/or physical activity intervention with a comparator group and targeted healthcare professionals. Comparator groups could include no intervention, usual care, active control or a waiting list group. Studies were required to report at least one diet, physical activity or weight-related outcome. There were no restrictions on intervention content, intervention duration or follow-up period. No language restrictions were applied.

### Data sources and search strategy

Seven electronic databases were searched from database inception (through to July 2012); Medline, Embase, DARE, PsycINFO, CINAHL, SPORTDiscus and the Cochrane Central Register of Controlled Trials (CENTRAL). The electronic search strategy (Additional file 1) was developed in Medline by combining a string of relevant Medical Subject Headings (MeSH) terms and text words. This was adapted between databases to allow for differences in accepted search terms and limits. Reference lists from retrieved primary studies and review articles were also searched.

### Study selection

To retrieve relevant studies, two reviewers independently screened all titles and abstracts generated from the searches. Studies identified as relevant or possibly relevant were obtained as full-text reports for further evaluation. Full texts were then examined by the same two reviewers for inclusion status. Reference lists of eligible studies and relevant review articles were also checked to identify additional applicable studies. Any disagreements on inclusion status were resolved through discussion with a third reviewer.

### Data extraction and quality assessment

For each study, data were extracted and study quality assessed by two reviewers using a structured proforma and cross-checked. Where any discrepancies were identified, they were resolved through discussion. The Effective Public Health Practice Project Quality Assessment Tool for Quantitative Studies was used to appraise the methodological rigour of all studies that met inclusion criteria. This tool has been determined suitable for use in systematic reviews of effectiveness [26] and has satisfactory content and construct validity [27,28].

Intervention and control group components of each study were also coded by two reviewers using guidance recommendations developed by NICE [29]. The components were coded as: behavioural, environmental, health check(s), incentive(s), active and continuous promotion of healthy choices, working practices and policies, supportive physical environment, recreational opportunities, informational and no intervention. If the intervention or control group components of a study could not be coded with any of these specified codes, they were coded as “other” with details provided. Discrepancies in coding were resolved by consensus or consultation with a third reviewer. In line with Taylor *et al*. [30], interventions were also coded based on the extent to which they were theory-based. Each study that utilised a theory to develop an intervention was also cross-tabulated with study quality and intervention effects on weight, dietary and physical activity related outcomes. This allowed the investigation of whether theory use in intervention development was associated with study quality and intervention effectiveness.

Data were extracted on diet-related, physical activity-related and weight-related outcome measures (where available). Where relevant data was not available in the published reports, efforts were made to contact study authors, or to calculate them directly from other data available in the paper e.g. from confidence intervals or p-values. Remaining missing standard deviations from individual studies that could not be retrieved from the study authors or calculated were imputed according to the methods described in the Cochrane Handbook for Systematic Reviews of Interventions [31,32]. Where data was imputed, this has been identified in the text. Any differences of opinion in data extraction or quality assessment of eligible studies were resolved by discussion, with reference to a third reviewer if no consensus could be reached.

### Data synthesis

As per our pre-specified protocol, studies were grouped according to length of follow-up (<12 months and ≥12 months) as we postulated *a priori* that the effects of the intervention would likely be different over the shorter and longer term. We had further proposed *a priori* to investigate the effect, if any, within timeframes of behavioural target (i.e. diet only, physical activity only or diet and physical activity combined). A narrative synthesis was used to analyse the key findings and meta-analysis undertaken where data allowed – in this case, meta-analysis could be undertaken for body weight data.

Study results of body weight outcomes were combined for meta-analysis using Review Manager (RevMan) V 5.2 software [33]. Two studies [34-36] included in the meta-analysis were cluster RCTs – neither of which had adjusted for clustering in their original analysis. In line with recommended methods [32], we calculated “effective sample sizes” for each study. This required the estimation of an intracluster correlation coefficient (ICC) for each study. As neither study had reported an ICC, we attempted to identify an appropriate ICC from other sources. Two previous estimates of ICC (0.007 and 0.00) for weight loss outcomes were identified [37,38]. As these estimates were very small we used a more conservative estimate of ICC (0.01) in our calculations, in line with wider published estimates of ICC for outcome measures [39]. Analysis was based on mean difference in body weight change between intervention and control groups. The meta-analysis adopted a random effects approach (most suitable when studies may be heterogeneous). For body weight data a weighted mean difference (WMD) was calculated (weighted by the inverse of the variance). Significance was set at *P* <0.05. For the test of heterogeneity, we used Higgins I^2^. Values >50% were taken to indicate that there was substantial statistical heterogeneity across studies [40].

### Sensitivity analysis

A sensitivity analysis was also carried out to evaluate the effect of RCT quality on intervention effect size.

### Assessment of publication bias

Publication bias was investigated using funnel plots [41]. We assessed funnel plot asymmetry visually with the Mean difference (MD) plotted against the MD standard error.

## Results

**Figure 1:**
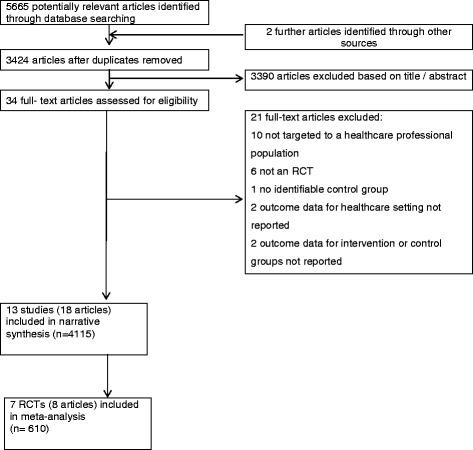
**Preferred reporting items for systematic reviews and meta-analyses (PRISMA) flow diagram for randomised controlled trials.**

The search identified 5665 potentially relevant articles. On further inspection, a total of 34 articles were retrieved for full text assessment. Overall, 13 studies met the inclusion criteria [34-36,42-57], of which seven (all those reporting body weight data) included data suitable for meta-analysis. The results of the literature search and the selection process are presented in Figure 1. Additional file 2 also provides a separate reference list of included and excluded studies.

### Study characteristics

Of the 13 studies which met the inclusion criteria, four were undertaken in the United Kingdom [42-45], two in Sweden [46,47], three in the United States of America (45, 46, 48); and one each in Australia [48], Norway [49], The Netherlands [55] and Denmark [36]. Nine studies were individually randomised controlled designs [34,42-46,52,53,55] and four used cluster randomised controlled designs [36,47,48,50]. A total of 3751 participants (1888 intervention, 1863 control) were recruited to the 13 studies. Intervention duration was less than 12 months in eight studies [34,42-45,52,53,55], and greater than or equal to 12 months in five studies [36,46-48,50]. Eight interventions assessed outcomes immediately after cessation of the intervention [43,45-48,53,55,57]. Only two studies were rated as ‘strong’ in methodological quality [36,45] with six of the studies scoring ‘moderate’ in quality [34,42,44,47,50,55] and the other five ‘weak’ [43,46,48,52,53]. Further intervention characteristics can be found in Additional file 3. Additional file 4 also provides detailed information about the quality assessment of each study.

### Results of individual studies

A summary of the reported effects of the 13 individual interventions included in this systematic review is presented in Table 1. Six interventions reported statistically significant effects on weight related outcomes, four interventions on dietary outcomes and six on physical activity outcomes. For BMI, body fat percentage, waist circumference, waist-hip ratio or diet and physical activity related outcomes sufficient data was not available for a pooled analysis. For instance, important data (e.g. confidence intervals, standard deviations) were not provided from primary papers and we were unable to obtain further information which precluded pooling of these outcomes.

**Table 1:** **Intervention effects, listed by behavioural target**

**Study**	**Behavioural target**	**Follow-up from baseline**	**Body weight**	**BMI**	**Body fat%**	**Waist circumference**	**Waist-hip ratio**	**Total energy intake**	**Total fat intake**	**Saturated fat intake**	**% Energy fat**	**Fruit intake**	**Vegetable intake**	**Fibre intake**	**Diet score**	**MPA**	**VPA**	**Total steps**	**Self-reported PA**
Barratt 1994 [52]	Diet	3 months	**↓**																
		6 months	**↑**					**↑**		**↓**	**↓**			**↑**					
Armitage 2001 [44]	Diet	5 months							**↑**	**↑**	**↓**								
Gamble 1993 [42]	Physical activity	11 weeks	**↓**		**↓**														
Gerdle 1995 [46]	Physical activity	12 months	**↓**																
Brox 2005 [53]	Physical activity	6 months																	**↑**
Hewitt 2008 [45]	Physical activity	4 weeks	**↓**	**↓**															
		8 weeks	**↓**	**↓**															
		12 weeks	**↓**	**↓**															
Von Thiel 2008 [47]	Physical activity	6 months																	**↑**
		12 months					**↑**												**↑**
Cockroft 1994 [43]	Diet and Physical activity	6 months		**↑**											**↓**				**↑**
Aldana 2005 [34]	Diet and Physical activity	6 weeks	**↑**	**↑**	**↑**			**↓**	**↑**	**↑**	**↑**	**↑**	**↑**	**↑**				**↑**	
		6 months	**↑**	**↑**	**↑**			**↑**	**↑**	**↑**	**↑**	**↑**	**↑**	**↑**				**↓**	
Racette 2009 [48]	Diet and Physical activity	6 months			**↓**	**↓**				**↑**		**↑**	**↑**						**↑**
		12 months	**↑**	**↑**						**↑**		**↑**	**↑**						**↑**
Lemon 2010 [50]	Diet and Physical activity	12 months		**↓**															
		24 months		**↓**															
Strijk 2012 [56]	Diet and Physical activity	6 months										**↑**				**↓**	**↓**		**↑**
Christensen 2011 [36]	Diet and Physical activity	3 months	**↑**	**↑**	**↑**	**↑**	**↓**												
		12 months	**↑**	**↑**	**↑**	**↑**													

*Abbreviations*: ↑ = Indicates a statistically significant effect in favour of the intervention; ↓ = Indicates a non-significant effect of intervention.

BMI = body mass index, MPA = Moderate physical activity, VPA = Vigorous physical activity, PA = Physical activity.

Blank cells = the outcome was not reported.

### Pooled estimate of body weight change

Data was available (or calculable) from all seven studies measuring change in body weight for inclusion in a meta-analysis. Data from these studies is presented in Table 2 with additional notes on calculations and imputations also included.

**Table 2:** **Body weight data from studies in meta-analysis and imputations of standard deviations for changes from baseline**

**Study**	**Follow-up time point (s)**	**Baseline weight (Kg) Intervention**	**Baseline weight (Kg) Control**	**Follow-up weight (Kg) Intervention**	**Follow-up weight (Kg) Control**	**Change weight (Kg) Intervention**	**Change weight (Kg) Control**	Difference change body weight (Kg) intervention Vs control*	**Quality Score**
**Behavioural target**									
				**n**	**n**	**n**	**n**		
		**Mean (SD)**	**Mean (SD)**	**Mean (SD)**	**Mean (SD)**	**Mean (SD** ^**a**^ **)**	**Mean (SD** ^**a**^ **)**	**Mean (95% CI)**	
Barratt 1994 [52]	6 months	71.0(NR)	70.4(NR)	70.6(NR)	70.5(NR)	−0.40(2.40^a b^)	+0.60(3.20^ab^)	−1.0 (−1.84, −0.16)	Weak
Dietary				56	130	56	130		
Gamble 1993 [42]	11 weeks	76.30(13.01)	78.90(5.14)	76.50(12.44)	79.10(5.38)	+0.20(4.80^c^)	+0.20(2.0^c^)	0 (−3.69, 3.69)	Moderate
Physical activity				8	6	8	6		
Hewitt 2008 [45]	8 weeks	68.5 (12.1)	66.4 (13.2)	69.4 (12.7^b^)	66.5 (13.0^b^)	+0.9 (4.68^c^)	+0.1 (4.90^c^)	0.8 (−3.51, 5.11)	Strong
Physical activity				12	8	12	8		
Gerdle 1995 [46]	12 months	67.0(11.6)	65.0(12.0)	66.0(10.9)	65.0(10.4)	−1.0(4.30^c^)	0.0(4.50^c^)	−1.0 (−2.99, 0.99)	Weak
Physical activity				32	45	32	45		
Aldana 2005 [34]	6 months	89.3(NR)	85.9(NR)	84.9(NR)	84.9(NR)	−4.4(5.05^d^)	−1.0(5.05^d^)	−3.4 (−5.06, −1.74)	Moderate
Dietary and Physical activity				64	79	64	79		
Racette 2009 [48]									
Dietary and Physical activity	12 months	92.4(24.9)	84.5(20.9)	91.6(25.5)	85.1(23.2)	−0.80(5.0^a^)	+0.60(5.0^a^)	−1.4 (−3.18, 0.38)	Weak
				42	34	42	34		
Christensen 2011 [36]	3 months	84.30(16.0)	83.0(14.4)	80.71(16.0)	83.68(14.4)	−3.59(3.80)	+0.68(2.37)	−4.27 (−5.53, −3.01)	Strong
Dietary and Physical activity				52	42	52	42		
	12 months	84.20(16.0)	83.0(14.4)	78.4(15.8)	82.7(14.6)	−5.80(5.90)	−0.30(5.40)	−5.50 (−7.79, −3.21)	
				52	42	52	42		

^a^SDs calculated directly from the reported confidence intervals; ^b^Additional unpublished data provided; ^c^Where change SDs were not explicitly reported, they were calculated from the reported baseline and final SDs using the correlation method advocated in the Cochrane handbook [32,33] – correlation coefficient calculated directly from the empirical data presented in Christensen [55]; ^d^SDs calculated directly from the reported p-value; NR = Not reported; *A minus figure indicates mean difference in body weight change in favour of intervention.

**Figure 2:**
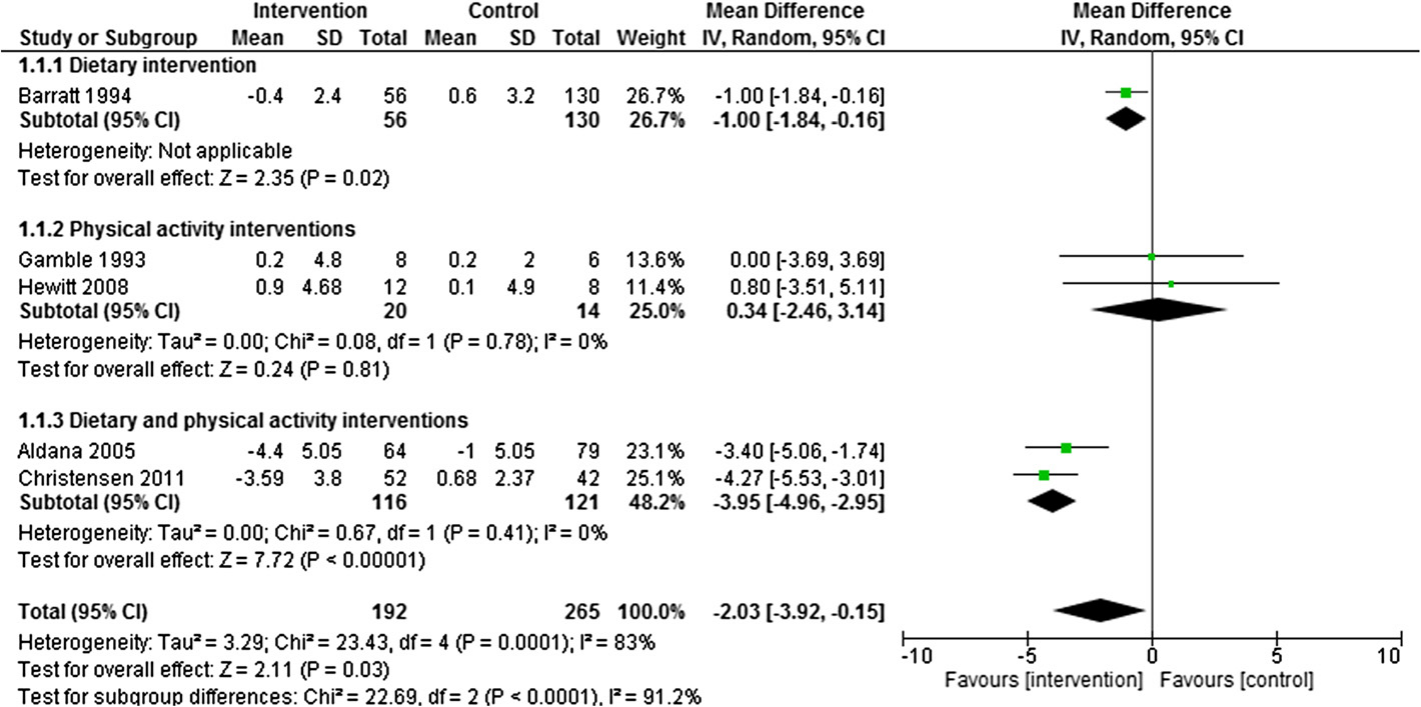
**Dietary, physical activity and dietary and physical activity interventions vs. control at <12 months follow-up.** Outcome: Body weight change (Kg).

Pooling results across the five studies which had follow-up under 12 months showed that there was a significantly greater reduction in body weight (−2.03 Kg, [95% CI −3.92 to - 0.15 Kg]) in participants allocated to some form of active intervention (diet only, physical activity only or dietary and physical activity combined interventions) compared with controls (Figure 2). However, there was evidence of significant heterogeneity across studies, and visual inspection of the forest plots indicates that interventions combining diet and physical activity achieved the largest reduction in body weight (−3.95 Kg, [95% CI −4.96 to −2.95 Kg]).

**Figure 3:**
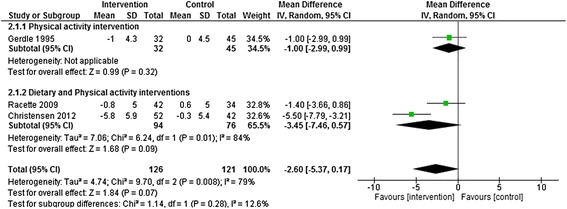
**Physical activity and dietary and physical activity interventions vs. control at > = 12 months follow-up.** Outcome: Body weight change (Kg).

There was no statistically significant difference in body weight change (−2.60 Kg, [95% CI – 5.37 to 0.17 Kg]) between intervention and control groups across the three studies with follow-up ≧12 months (Figure 3). Again, however, there was evidence of significant heterogeneity across studies, with diet and physical interventions again showing the largest effects.

### Sensitivity analysis

**Figure 4:**
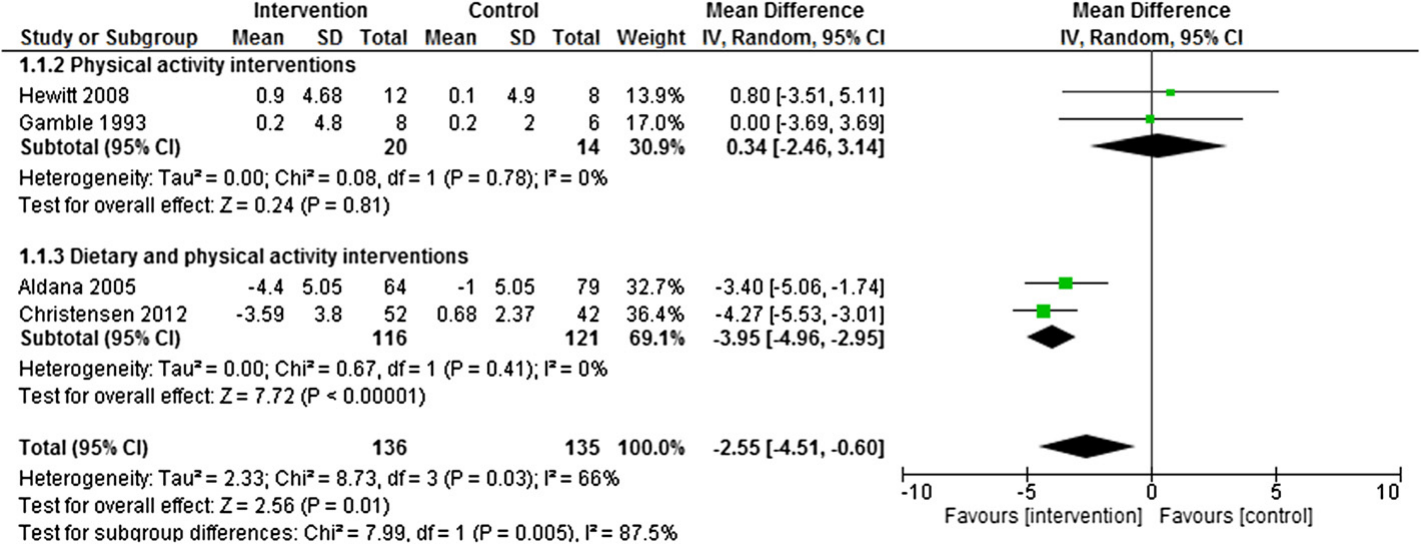
**Sensitivity analysis interventions vs. control at <12 months follow-up.** Outcome: Body weight change (Kg).

One of the five studies which had follow-up <12 months was rated as weak in methodological quality. Removing this study resulted in a pooled effect size of −2.55 Kg (4 studies – 2 Strong quality, 2 Moderate quality; [95% CI – 4.51 to – 0.60 Kg] in 271 healthcare professionals) (Figure 4). Results suggest that excluding the weak quality study had no statistical effects on pooled effect sizes. There were insufficient studies to allow sensitivity analysis of studies with a follow up ≧12 months.

### Analysis of publication bias

**Figure 5:**
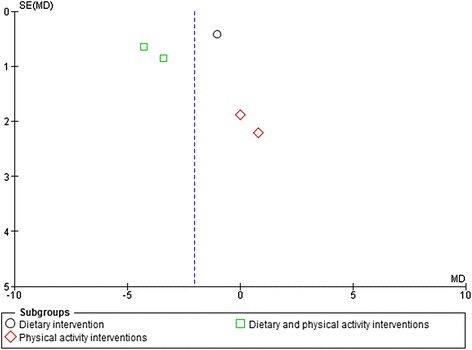
**Funnel plot of dietary, physical activity and dietary and physical activity interventions vs. control at <12 months follow-up.** Outcome: Body weight change (Kg).

**Figure 6:**
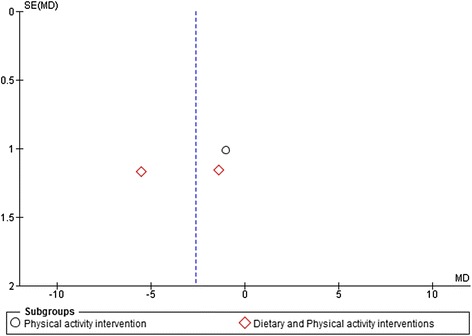
**Funnel plot of physical activity and dietary and physical activity interventions vs. control at > = 12 months follow-up.** Outcome: Body weight change (Kg).

Visual inspection of the funnel plot across the five studies which had follow-up <12 months (Figure 5) suggests some asymmetry, indicating potential publication bias. However, there were too few studies available to formally assess this. The funnel plot across the three studies with follow-up ≧12 months was inconclusive due to a small number of studies available (Figure 6).

### Intervention effects by intervention components

All interventions included multiple distinguishable components (e.g. information provision, structured exercise, diet diaries, etc.) but there were no consistent differences apparent in the components of effective and non-effective interventions.

### Intervention effects by theoretical basis of intervention

Four studies reported using theory to guide workplace intervention development [44,48,50,55]. Of these, three studies broadly described how theory was used to inform intervention design but it was unclear how theory was operationalised in any of these interventions. Two of these studies named the theory used as The Trans Theoretical Model of Behavioural Change [44,48]. The third study named and utilised several different theories including Diffusion of Innovation Theory, the Ecologic Conceptual Framework, the Health Belief Model, Organizational Development Theory, Social Cognitive Theory and the Theory of Reasoned Action [50]. One further study described how theoretical constructs were used to inform the design of each specific component of the intervention but did not reference any specific theory used [55]. Table 3 outlines the reported theoretical basis of these interventions along with their methodological quality and effects on weight, dietary and physical activity outcomes. Of the seven studies evaluating intervention effects on a weight related outcome, 50% of the interventions reporting a theoretical basis were effective, compared with 57% of interventions not reporting a theory base. For physical activity related outcomes, we found no difference in effectiveness for theory-based interventions compared with interventions not reporting a theory base (100% effective versus 100% effective). For dietary related outcomes, 100% of the theory-based interventions were effective, compared with 50% of interventions not reporting a theory base. The two studies rated as ‘strong’ in methodological quality [36,45] were of interventions not reported to be based on theory. Of the six studies scoring ‘moderate’ in quality [34,42,44,47,50,55], three evaluated a theory-based intervention and three were of interventions not reported to be based on theory. Four of the five studies rated as ‘weak’ were of interventions not reporting a theory base [43,46,48,52,53].

**Table 3:** **Intervention effects, listed by theoretical basis**

**Study**	**Behaviour change theory/theories used to** develop intervention*	**Study quality score**	**Follow-up from baseline**	**Body weight**	**BMI**	**Body fat%**	**Waist circumference**	**Waist-hip ratio**	**Total energy intake**	**Total fat intake**	**Saturated fat intake**	**% Energy fat**	**Fruit intake**	**Vegetable intake**	**Fibre intake**	**Diet score**	**MPA**	**VPA**	**Total steps**	**Self-reported PA**
Armitage 2001 [44]	The Trans Theoretical Model of Behavioural Change	Moderate	5 months							**↑**	**↑**	**↓**								
Racette 2009 [48]	The Trans Theoretical Model of Behavioural Change	Moderate	6 months			**↓**	**↓**				**↑**		**↑**	**↑**						**↑**
			12 months	**↑**	**↑**						**↑**		**↑**	**↑**						**↑**
Lemon 2010 [50]	Diffusion of Innovation Theory, the Ecologic Conceptual Framework, the Health Belief Model, Organizational Development Theory, Social Cognitive Theory and the Theory of Reasoned Action	Moderate	12 months		**↓**															
			24 months		**↓**															
Strijk 2012 [56]	Study used theoretical constructs to inform the design of each specific component of the intervention but did not reference any specific theory used	Weak	6 months										**↑**				**↓**	**↓**		**↑**
Gamble 1993 [42]	No theory reported	Moderate	11 weeks	**↓**		**↓**														
Barratt 1994 [52]	No theory reported	Weak	3 months	**↓**																
			6 months	**↑**					**↑**		**↓**	**↓**			**↑**					
Cockroft 1994 [43]	No theory reported	Weak	6 months		**↑**											**↓**				**↑**
Gerdle 1995 [46]	No theory reported	Weak	12 months	**↓**																
Aldana 2005 [34]	No theory reported	Moderate	6 weeks	**↑**	**↑**	**↑**			**↓**	**↑**	**↑**	**↑**	**↑**	**↑**	**↑**				**↑**	
			6 months	**↑**	**↑**	**↑**			**↑**	**↑**	**↑**	**↑**	**↑**	**↑**	**↑**				**↓**	
Brox 2005 [53]	No theory reported	Weak	6 months																	**↑**
Von Thiel Schwarz 2008 [47]	No theory reported	Moderate	6 months																	**↑**
			12 months					**↑**												**↑**
Hewitt 2008 [45]	No theory reported	Strong	4 weeks	**↓**	**↓**															
			8 weeks	**↓**	**↓**															
			12 weeks	**↓**	**↓**															
Christensen 2011 [36]	No theory reported	Strong	3 months	**↑**	**↑**	**↑**	**↑**	**↓**												
			12 months	**↑**	**↑**	**↑**	**↑**													

*Reported by study authors; ↑ = Indicates a statistically significant effect in favour of the intervention; ↓ = Indicates a non-significant effect of intervention; BMI = body mass index, MPA = Moderate physical activity, VPA = Vigorous physical activity, PA = Physical activity; Blank cells = the outcome was not reported.

## Discussion

This systematic review and meta-analysis synthesised the best available RCT evidence of workplace-based dietary and physical activity interventions specifically targeted to healthcare professionals. Thirteen RCT studies were identified; nine of these reporting statistically significant (between-group) differences in either dietary, physical activity or weight-related outcomes.

The results of the meta-analysis showed that workplace interventions which targeted both diet and physical activity resulted in the largest observed differences in weight reduction (2 studies – 1 Strong quality, 1 Moderate quality; −3.95 Kg, [95% CI – 4.96 to – 2.95] in 237 healthcare professionals up to 12 months of follow-up). This effect is larger than those reported in previous meta-analyses of workplace-based interventions (those reviews had not however focused on healthcare professionals). For instance, Anderson *et al*. pooled data from RCTs with 6–12 months of follow-up and reported effect estimates of −1.3 kg (nine studies; [95% CI −2.1 to −0.45]) [58]. Further, Ver Weij *et al*. demonstrated that combined dietary and physical activity interventions over 8 weeks-18 months of follow-up resulted in modest weight loss of −1.19 kg (nine studies; [95% CI −1.64 to −0.74]) [59].

There are a number of possible explanations for these differences. One possible reason for the larger effect size observed in our meta-analysis is that healthcare professionals may be more amenable to behaviour change than the general population samples included in previous meta-analyses resulting in greater weight loss. For instance, Anderson et al. included studies focusing on varied occupational groups such as policemen and male employees working for a building maintenance company [58]. Further, it has been reported that small studies included in a meta-analysis tend to produce larger intervention effect sizes than larger studies [60]. Therefore, the small sample sizes of studies included in our meta-analysis may be a potential reason for the differences in findings observed. As Ver Weij *et al*. excluded interventions aimed solely at overweight subjects as well as weight loss interventions a possible explanation could be that interventions aimed specifically at weight loss and/or where the targeted participants are overweight/obese may be more effective compared with interventions without a specific weight-related aim. Future research should investigate whether studies with specific weight-related aims produce greater weight loss than those without specific weight-related aims. Notwithstanding this, conclusions regarding the effectiveness of dietary and physical activity combined interventions on healthcare professional body weight are limited because of the small number of studies and small sample sizes. Therefore, these preliminary findings require confirmation by further RCTs with larger sample sizes.

The findings of individual trials not included in the meta-analysis also merit mention. These trials demonstrated significant effects on a range of dietary intake outcomes such as reducing total fat and saturated fat intake and increasing fibre and fruit intake. Significant effects on physical activity outcomes were also reported. However, a variety of different methods were used across studies to measure dietary and physical activity behaviours, precluding cumulative analysis. For example, of the two studies measuring fruit intake [34,48], one study used a Block 98 full-length dietary questionnaire [34] while the other study used the National Institutes of Health Fruit and Vegetable Screener [48]. The methods used in the included studies to measure physical activity outcomes also varied and included the use of the International Physical Activity Questionnaire (IPAQ) [48], a 7-day self-reported pedometer log [34] and an accelerometer [56]. In addition, units of measure were heterogeneous thus precluding pooling of this data from individual studies. For instance, effects on saturated fat intake were reported as mean grams per day [44], % total energy [34], and intake of saturated fat, fatty meats, and fried foods [48]. Similarly, physical activity was reported as exercise frequency per month [43], self-rated physical activity (aerobics, running, and swimming) hours per week [47], total steps per week [34] and moderate-vigorous physical activity minutes per week [54].

Two studies also conducted process evaluations [34,53]. Reasons why an effective intervention works/could be improved or why interventions are ineffective/ have unexpected consequences can be better understood through the undertaking of a process evaluation [61,62]. For instance, in their process evaluation Lemon and colleagues revealed a dose–response relationship between BMI reduction and intervention participation, with effectiveness proportional to the extent of active participation [34]. The authors also report that the planned placement of healthy eating options in cafeterias was not fully implemented as intended and that vending machine modifications were not implemented at all [34]. Given this, researchers developing future workplace interventions targeted to healthcare professionals should consider including a process evaluation nested within an intervention in order to delineate causal mechanisms, identify contextual factors related to variations in outcomes, and to determine the fidelity and quality of intervention implementation [63].

A key finding of this systematic review is that the majority of workplace dietary and physical interventions targeting healthcare professionals did not report their theoretical development. The use of theory to develop interventions, and adequate reporting of this, facilitates investigators in measuring and describing causal pathways through which targeted behaviour change occurs. Therefore, failure to report the theoretical underpinning of interventions hampers our understanding of the mechanisms involved in behaviour change [59]. Only four studies reported the theoretical basis of their intervention, precluding formal comparison of theoretically derived interventions with others. A qualitative comparison suggested that interventions based on theory were not different in terms of effectiveness, than those interventions that were not based on theory. These findings are inconsistent with evidence from earlier reviews that argued that theoretically underpinning interventions enhances intervention effectiveness [64,65]. However, as study heterogeneity (in terms of theories and outcomes used) precluded a formal comparison of theoretically derived interventions with others, firm conclusions about the utility of underpinning interventions with theory in this context cannot be drawn.

Most of the interventions in this review were not reported in sufficient detail to allow analysis of specific individual intervention components. In other words, it was not possible to identify the ‘active ingredients’ in successful interventions as it was unclear what behavioural strategies or combination of behavioural strategies produced the most consistent effects for dietary and physical activity behaviour change and weight loss in healthcare professionals. Moreover, details of intervention characteristics such as intervention delivery source, delivery mode (group, individual or both), and intervention contact time were not reported in detail, limiting this review’s capacity to elucidate their role in intervention effectiveness. There is emerging evidence that the active content of a control group can explain an intervention’s effects [66]. For example, a recent systematic review and meta-regression of behavioural weight loss interventions found control groups receiving active content such as education sessions or advice to maintain usual behaviour patterns lost significantly more weight than no intervention control groups (1.23 Kg weight loss compared to no weight change respectively) [67]. Therefore, it is worth noting that six of the thirteen included trials in this review contained an active control group. However, similar to intervention group descriptions the information provided on control group conditions lacked detail.

Workplace dietary and physical activity interventions are complex and their implementation involves a number of detailed steps. It is possible that the complexity of these interventions coupled with journal word limit requirements could account for studies not being able to provide sufficient details on intervention and control group content. In order to address this, trial authors should consider reporting interventions in line with the *recommendations to Improve Reporting of the Content of Behaviour Change Interventions* set out by the Workgroup for Intervention Development and Evaluation Research (WIDER) [68] and the Consolidated Standards of Reporting Trials (CONSORT) guidance for non- pharmacological interventions [69]. Trial authors should also report the trial register number and refer to the published study protocol where possible when publishing findings. In addition, systematic review authors should endeavour to consult trial register entries and/or obtain the study protocol publication where more space is available to report details on intervention and control group content. Freedland and colleagues also suggest developing an evidence-based scientific consensus statement or guideline on control groups in behavioural RCTs [70]. Moreover, future studies may benefit from exploring ways in which each component can be given sufficient attention and focus, perhaps by implementing them in a phased manner. To identify ‘active ingredients’, clear specification of the behaviour change techniques (BCTs) used in interventions has also been advocated [71]. This in turn may permit more rigorous evaluation of future interventions through the application of a reliable healthy eating and physical activity BCT coding scheme such as the 40 item taxonomy of behaviour change techniques developed by Michie *et al*. [72].

It is important to emphasise that, although the evidence regarding the effectiveness of dietary and/or physical activity interventions for changing weight, dietary and physical activity outcomes in healthcare professionals is currently limited, it does not mean that there is evidence of no effect from the interventions. Additionally, these interventions are unlikely to cause any harm. We therefore tentatively recommend such interventions may be beneficial in practice and should be offered to healthcare professionals. The potential number of healthcare professionals who may benefit from participation lends further support for this recommendation. Until further robust evidence is available, practitioners and decision-makers should continue to use current clinical practice guidelines, which outline various workplace intervention options and approaches.

### Strengths of systematic review

To our knowledge, this is the first review to examine the effects of workplace-based dietary and/or physical activity interventions in the wider healthcare professional population. As such, this review makes a valuable contribution to this area of research. We used explicit methods to code all intervention and control group components and reviewer errors and bias were minimised at the title and abstract screening, quality assessment and data extraction stages due to a minimum of two reviewers performing these tasks. Efforts were made to retrieve missing data through direct contact with authors. This review extends the evidence from a recent meta-analysis of interventions conducted across heterogeneous workplaces [59] and one recent systematic review [21] of non-RCTs of workplace-based interventions for the nursing workforce. Seven of the studies in our review were not included in these previous reviews.

### Limitations of systematic review

Direct comparison between intervention components across studies and specific outcomes was difficult due to the disparate nature of interventions and the limited number of interventions reporting similar outcomes. It should be considered that publication bias may have impacted on the reporting of studies as relevant studies may not be published [73]. The funnel plot of interventions with follow up <12 months indicates the possible absence of studies. However, publication bias assessed through visual inspection of a funnel plot for asymmetry is subject to inconsistency, with ≥10 studies being required to differentiate real from spurious asymmetry [32]. Although a validated quality assessment tool was used in duplicate to assess RCT quality, other available tools may have reached different conclusions on the overall quality of the same RCTs. In addition, our meta-analysis findings are based on a limited number of primary RCTs. Whilst not unusual - the median number of RCTs included in formal meta-analyses in reviews within the Cochrane library is three [74] – the limitations of meta-analysis based on limited numbers of RCTs and limited sample sizes should not be ignored.

## Conclusions

Our review suggests workplace-based dietary and physical interventions may be potentially effective in reducing weight and changing the dietary and physical activity behaviours of healthcare professionals. However the available evidence base is limited with several important gaps identified which cause uncertainty in establishing what intervention content and characteristics contribute most to intervention effectiveness. As part of a research agenda to improve the interpretation of future interventions, further studies are required that: (i) publish comprehensive descriptive information on intervention content (ii) code effective intervention and control group components in workplace dietary and physical activity interventions targeted to healthcare professionals using a standardised taxonomy of BCTs and (iii) contain a minimum follow-up of 12 months in order to clarify the sustainability of intervention effects. Future interventions underpinned by psychological theory and providing details of how the selected theory is proposed to function within the intervention are also needed to enhance our understanding of the mechanisms behind intervention effects.

## Additional files

Additional file 1:
**Medline (Ovid) search strategy.**
Additional file 2:
**Reference list of included and excluded studies.**
Additional file 3:
**Characteristics of included interventions (stratified by behavioural target).**
Additional file 4:
**Quality assessment of included randomised controlled trials (structured by year of publication).**

## References

[1] Pate RR, Pratt M, Blair SN, Haskell WL, Macera CA, Bouchard C, Buchner D, Ettinger W, Heath GW, King AC, Kriska A, Leon AS, Marcus BS, Morris J, Paffenbarger RS, Patrick K, Pollock ML, Rippe JM, Sallis J, Wilmore JH (1995). Physical activity and public health: a recommendation from the Centers for Disease Control and Prevention and the American College of Sports Medicine. JAMA.

[2] Cross-Government Obesity Unit: **Healthy Weight, Healthy Lives: A Cross-Government Strategy for England**. 2008. [WWW document]. URL http://webarchive.nationalarchives.gov.uk/20100407220245/http://www.dh.gov.uk/en/Publicationsandstatistics/Publications/PublicationsPolicyAndGuidance/DH_082378.

[3] Department of Health: **Healthy Weight, Healthy Lives: One Year On**. 2009. [WWW document]. URL http://webarchive.nationalarchives.gov.uk/20100407220245/http://www.dh.gov.uk/en/Publicationsandstatistics/Publications/PublicationsPolicyAndGuidance/DH_097523.

[4] Blake H, Mo PKH, Lee S, Batt ME (2012). Health in the NHS: lifestyle behaviours of hospital employees. Perspect Public Health.

[5] Pohjonen T (2001). Age-related physical fitness and the predictive values of fitness tests for work ability in home care work. J Occup Environ Med.

[6] Zapka JM, Lemon SC, Magner RP, Hale J (2009). Lifestyle behaviours and weight among hospital-based nurses. J Nurs Manag.

[7] Han K, Trinkoff AM, Storr CL, Geiger-Brown J (2011). Job stress and work schedules in relation to nurse obesity. J Nurs Adm.

[8] Bogossian FE, Hepworth J, Leong GM, Flaws DF, Gibbons KS, Benefer CA, Turner CT (2012). A cross-sectional analysis of patterns of obesity in a cohort of working nurses and midwives in Australia, New Zealand, and the United Kingdom. Int J Nurs Stud.

[9] Cheung ST (2003). The effects of chocolates given by patients on the well-being of nurses and their support staff. Nutr Health.

[10] Jinks AM, Lawson V, Daniels R (2003). A survey of the health needs of hospital staff: Implications for health care managers. J Nurs Manag.

[11] Humphreys SL (2007). Obesity in patients and nurses increases the nurse’s risk of injury lifting patients. Bariatr Nurs Surg Patient Care.

[12] Østbye T, Dement JM, Krause KM (2007). Obesity and workers’ compensation: results from the duke health and safety surveillance system. Arch Intern Med.

[13] Ferrie JE, Head J, Shipley MJ, Vahtera J, Marmot MG, Kivimäki M (2007). BMI, obesity, and sickness absence in the Whitehall II study. Obesity.

[14] Harvey SB, Glozier N, Carlton O, Mykletun A, Henderson M, Hotopf M, Holland-Elliot K (2010). Obesity and sickness absence: Results from the CHAP study. Occup Med.

[15] Goetzel RZ, Gibson TB, Short ME, Chu BC, Waddell J, Bowen J, Lemon SC, Fernandez ID, Ozminkowski RJ, Wilson MG, DeJoy DM (2010). A multi-worksite analysis of the relationships among body mass index, medical utilization, and worker productivity. J Occup Environ Med.

[16] Blaber AY (2005). Exercise: who needs it?. Br J Nurs.

[17] Davey MM, Cummings G, Newburn-Cook CV, Lo EA (2009). Predictors of nurse absenteeism in hospitals: a systematic review. J Nurs Manag.

[18] Zhu D, Norman IJ, While AE (2011). The relationship between health professionals’ weight status and attitudes towards weight management: a systematic review. Obes Rev.

[19] Katz DL, O'Connell M, Yeh MC, Nawaz H, Njike V, Anderson LM, Cory S, Dietz W, Task Force on Community Preventive Services (2005). Public health strategies for preventing and controlling overweight and obesity in school and worksite settings: a report on recommendations of the Task Force on Community Preventive Services. MMWR Recomm Rep.

[20] Smedslund G, Fisher KJ, Boles SM, Lichtenstein E (2004). The effectiveness of workplace smoking cessation programmes: a meta-analysis of recent studies. Tob Control.

[21] Chan CW, Perry L (2012). Lifestyle health promotion interventions for the nursing workforce: a systematic review. J Clin Nurs.

[22] Jemmott JB, Jemmott LS (2000). HIV risk reduction behavioral interventions with heterosexual adolescents. AIDS.

[23] Michie S, Abraham C (2004). Interventions to change health behaviours: evidence based or evidence inspired. Psychol Health.

[24] Shrout PE, Bolger N (2002). Mediation in experimental and nonexperimental studies: New procedures and recommendations. Psychol Methods.

[25] Liberati A, Altman DG, Tetzlaff J, Mulrow C, Gøtzsche PC, Ioannidis JP, Clarke M, Devereaux PJ, Kleijnen J, Moher D (2009). The PRISMA statement for reporting systematic reviews and meta-analyses of studies that evaluate healthcare interventions: explanation and elaboration. BMJ.

[26] Deeks JJ, Dinnes J, D'Amico R, Sowden AJ, Sakarovitch C, Song F, Petticrew M, Altman DG, International Stroke Trial Collaborative Group, European Carotid Surgery Trial Collaborative Group (2003). Evaluating non-randomised intervention studies. Health Technol Assess.

[27] Jackson N, Waters E (2005). Criteria for the systematic review of health promotion and public health interventions. Health Promot Int.

[28] Thomas BH, Ciliska D, Dobbins M, Micucci S (2004). A process for systematically reviewing the literature: providing the research evidence for public health nursing interventions. Worldviews Evid Based Nurs.

[29] National Institute for Health and Clinical Excellence (NICE) (2006). Obesity: The Prevention, Identification, Assessment and Management of Overweight and Obesity in Adults and Children. Clinical guideline. CG43.

[30] Taylor N, Conner M, Lawton R (2012). The impact of theory on the effectiveness of worksite physical activity interventions: a meta-analysis and meta-regression. Health Psychol Rev.

[31] Deeks JJ, Higgins JPT, Altman DG (eds): **Chapter 9: Analysing data and undertaking meta-analyses. In: Higgins JPT, Green S (eds). Cochrane Handbook for Systematic Reviews of Interventions Version 5.0.1 (updated September 2009).** The Cochrane Collaboration, 2009. Available from http://handbook.cochrane.org/.

[32] Higgins JP, Deeks JJ, Altman DG: **Chapter 16: Special topics in statistics. In: Higgins JPT, Green S (eds). Cochrane handbook for systematic reviews of interventions Version 5.0.2 [updated September 2009].** The Cochrane Collaboration, 2009. Available from www.cochrane-handbook.org

[33] Cochrane Collaboration (2012). Review Manager (RevMan) [Computer Program]. Version 5.2.

[34] Aldana SG, Greenlaw RL, Diehl HA, Salberg A, Merrill RM, Ohmine S (2005). The effects of a worksite chronic disease prevention program. J Occup Environ Med.

[35] Holtermann A, Jørgensen MB, Gram B, Christensen JR, Faber A, Overgaard K, Ektor-Andersen J, Mortensen OS, Sjøgaard G, Søgaard K (2010). Worksite interventions for preventing physical deterioration among employees in job-groups with high physical work demands: Background, design and conceptual model of FINALE. BMC Public Health.

[36] Christensen JR, Faber A, Ekner D, Overgaard K, Holtermann A, Søgaard K (2011). Diet, physical exercise and cognitive behavioral training as a combined workplace based intervention to reduce body weight and increase physical capacity in health care workers - A randomized controlled trial. BMC Public Health.

[37] Wing RR, Leahey T, Jeffery R, Johnson KC, Hill JO, Coday M, Espeland MA, Look AHEAD Research Group (2014). Do weight loss and adherence cluster within behavioral treatment groups?. Obesity (Silver Spring).

[38] Simon GE, Rhode P, Ludman EJ, Jeffery RW, Linde JA, Operskalski BH, Arterburn D, Finch EA (2010). Is success in weight loss treatment contagious (Do attendance and outcomes cluster within treatment groups)?. Obes Res Clin Pract.

[39] Campbell MK, Fayers PM, Grimshaw JM (2005). Determinants of the intracluster correlation coefficient in cluster randomised trials. Clin Trials.

[40] Higgins JP, Thompson SG, Deeks JJ, Altman DG (2003). Measuring inconsistency in meta-analysis. BMJ.

[41] Sterne JAC, Sutton AJ, Ioannidis JP, Terrin N, Jones DR, Lau J, Carpenter J, Rücker G, Harbord RM, Schmid CH, Tetzlaff J, Deeks JJ, Peters J, Macaskill P, Schwarzer G, Duval S, Altman DG, Moher D, Higgins JP (2011). Recommendations for examining and interpreting funnel plot asymmetry in meta-analyses of randomised controlled trials. BMJ.

[42] Gamble RP, Boreham CAG, Stevens AB (1993). Effects of a 10-week exercise intervention programme on exercise and work capacities in Belfast’s ambulancemen. Occup Med.

[43] Cockcroft A, Gooch C, Ellinghouse C, Johnston M, Michie S (1994). Evaluation of a programme of health measurements and advice among hospital staff. Occup Med.

[44] Armitage CJ, Conner M (2001). Efficacy of a minimal intervention to reduce fat intake. Soc Sci Med.

[45] Hewitt JA, Whyte GP, Moreton M, Van Someren KA, Levine TS (2008). The effects of a graduated aerobic exercise programme on cardiovascular disease risk factors in the NHS workplace: A randomised controlled trial. J Occup Med Toxicol.

[46] Gerdle B, Brulin C, Elert J, Eliasson P, Granlund B (1995). Effect of a general fitness program on musculoskeletal symptoms, clinical status, physiological capacity, and perceived work environment among home care service personnel. J Occup Rehab.

[47] von Thiele Schwarz U, Lindfors P, Lundberg U (2008). Health-related effects of worksite interventions involving physical exercise and reduced workhours. Scand J Work Environ Health.

[48] Racette SB, Deusinger SS, Inman CL, Burlis TL, Highstein GR, Buskirk TD, Steger-May K, Peterson LR (2009). Worksite Opportunities for Wellness (WOW): Effects on cardiovascular disease risk factors after 1 year. Prev Med.

[49] Zapka J, Lemon SC, Estabrook BB, Jolicoeur DG (2007). Keeping a step ahead: Formative phase of a workplace intervention trial to prevent obesity. Obesity.

[50] Lemon SC, Zapka J, Li W, Estabrook B, Rosal M, Magner R, Andersen V, Borg A, Hale J (2010). Step ahead. A worksite obesity prevention trial among hospital employees. Am J Prev Med.

[51] Estabrook B, Zapka J, Lemon SC (2012). Evaluating the implementation of a hospital work-site obesity prevention intervention: Applying the re-aim framework. Health Promot Pract.

[52] Barratt A, Reznik R, Irwig L, Cuff A, Simpson JM, Oldenburg B, Horvath J, Sullivan D (1994). Work-site cholesterol screening and dietary intervention: The Staff Healthy Heart Project. Am J Public Health.

[53] Brox JI, Froøystein O (2005). Health-related quality of life and sickness absence in community nursing home employees: Randomized controlled trial of physical exercise. Occup Med.

[54] Strijk JE, Proper KI, Van Der Beek AJ, Van Mechelen W (2009). The vital@work study. The systematic development of a lifestyle intervention to improve older workers’ vitality and the design of a randomised controlled trial evaluating this intervention. BMC Public Health.

[55] Strijk JE, Proper KI, van der Beek AJ, van Mechelen W (2011). A process evaluation of a worksite vitality intervention among ageing hospital workers. Int J Behav Nutr Phys Act.

[56] Strijk JE, Proper KI, Van der Beek AJ, van Mechelen W (2012). A worksite vitality intervention to improve older workers’ lifestyle and vitality-related outcomes: Results of a randomised controlled trial. J Epidemiol Community Health.

[57] Christensen JR, Overgaard K, Carneiro IG, Holtermann A, Søgaard K (2012). Weight loss among female health care workers-a 1-year workplace based randomized controlled trial in the FINALE-health study. BMC Public Health.

[58] Anderson LM, Quinn TA, Glanz K, Ramirez G, Kahwati LC, Johnson DB, Buchanan LR, Archer WR, Chattopadhyay S, Kalra GP, Katz DL, Task Force on Community Preventive Services (2009). The effectiveness of worksite nutrition and physical activity interventions for controlling employee overweight and obesity a systematic review. Am J Prev Med.

[59] Verweij LM, Coffeng J, van Mechelen W, Proper KI (2011). Meta-analyses of workplace physical activity and dietary behaviour interventions on weight outcomes. Obes Rev.

[60] Sterne JAC, Gavaghan D, Egger M (2000). Publication and related bias in meta-analysis: power of statistical tests and prevalence in the literature. J Clin Epidemiol.

[61] Begg C, Cho M, Eastwood S, Horton R, Moher D, Olkin I, Pitkin R, Rennie D, Schulz KF, Simel D, Stroup DF (1996). Improving the quality of reporting of randomized controlled trials. The CONSORT statement. JAMA.

[62] Rychetnik L, Frommer M, Hawe P, Shiell A (2002). Criteria for evaluating evidence on public health interventions. J Epidemiol Community Health.

[63] Craig P, Dieppe P, Macintyre S, Michie S, Nazareth I, Petticrew M (2008). Developing and Evaluating Complex Interventions.

[64] Albarracin D, Gillette JG, Earl AN, Glasman LR, Durantini MR, Ho MH (2005). A test of major assumptions about behavior change: a comprehensive look at the effects of passive and active HIV-prevention interventions since the beginning of the epidemic. Psychol Bull.

[65] Noar SM, Zimmerman RS (2005). Health behavior theory and cumulative knowledge regarding health behaviours: are we moving in the right direction?. Health Educ Res.

[66] de Bruin M, Viechtbauer W, Schaalma HP, Kok G, Abraham C, Hospers HJ (2010). Standard care impact on effects of highly active antiretroviral therapy adherence interventions: A meta-analysis of randomized controlled trials. Arch Intern Med.

[67] Waters L, St George A, Chey T, Bauman A (2012). Weight change in control group participants in behavioural weight loss interventions: A systematic review and meta-regression study. BMC Med Res Methodol.

[68] Workgroup for Intervention Development and Evaluation Research: **WIDER recommendations**. [http://www.equator-network.org/reporting-guidelines/wider-recommendations-for-reporting-of-behaviour-change-interventions/] 10.1186/1748-5908-8-52 PMC3661354 23680355

[69] Boutron I, Moher D, Altman DG, Schulz KF, Ravaud P (2008). Extending the CONSORT statement to randomized trials of nonpharmacologic treatment: explanation and elaboration. Ann Intern Med.

[70] Freedland KE, Mohr DC, Davidson KW, Schwartz JE (2011). Usual and unusual care: Existing practice control groups in randomized controlled trials of behavioural interventions. Psychosom Med.

[71] Michie S, Johnston M (2012). Theories and techniques of behaviour change: Developing a cumulative science of behaviour change. Health Psychol Rev.

[72] Michie S, Ashford S, Sniehotta FF, Dombrowski SU, Bishop A, French DP (2011). A refined taxonomy of behaviour change techniques to help people change their physical activity and healthy eating behaviours: The CALO-RE taxonomy. Psychol Health.

[73] Sutton AJ, Duval SJ, Tweedie RL, Abrams KR, Jones DR (2000). Empirical assessment of effect of publication bias on meta-analyses. BMJ.

[74] Davey J, Turner RM, Clarke MJ, Higgins JPT (2011). Characteristics of meta-analyses and their component studies in the Cochrane Database of Systematic Reviews: a cross-sectional, descriptive analysis. BMC Med Res Methodol.

