# Supplementary material for: Effects of workplace-based dietary and/or physical activity interventions for weight management targeting healthcare professionals: a systematic review of randomised controlled trials
Source: BMC Obes. 2014 Nov 14;1:23. doi: 10.1186/s40608-014-0023-3 (PMC4511014; doi:10.1186/s40608-014-0023-3)
Supplement: Additional file 1: — Medline (Ovid) search strategy. [file 40608_2014_23_MOESM1_ESM.docx]

**Additional file 1: Medline (Ovid) search strategy**

1. (work place* or workplace* or work site* or work location* or work setting*).ti.

2. exp *health personnel/

3. ((health or healthcare or health care or medical or hospital or allied health) adj1 (personnel or worker* or provider* or employee* or staff or professional*)).tw.

4. occupational therapist*.tw.

5. (physiotherapist* or physical therapist*).tw.

6. (paramedic* or ambulance person* or ambulance staff or emergency person* or emergency staff).tw.

7. (dieti*ian or nutritionist*).tw.

8. pharmacist*.tw.

9. (nursing adj1 (personnel or staff)).tw.

10. (nurse* or doctor* or physician* or clinician* or surgeon* or general practitioner* or gp or gps).tw.

11. or/1-10

12. intervention?.tw.

13. exp Exercise/

14. exercise*.tw.

15. (physical* adj1 activit*).tw.

16. diet, carbohydrate-restricted/ or diet, fat-restricted/ or diet, reducing/ or exp energy intake/

17. ((diet or diets) adj1 (fat restrict* or fat reduc* or carbohydrate restrict* or low carb* or low calorie* or calorie restrict*)).tw.

18. or/12-17

19. Physical Fitness/

20. aerobic capacity.tw.

21. ((increas* or improv*) adj1 (fitness or physical activity)).tw.

22. Food habits/ or Food preferences/

23. ((fruit* or vegetable* or energy) adj1 (intak* or consumed or consume or consumes or consumption)).tw.

24. (dietary adj1 (intake or behavio*r*)).tw.

25. Obesity/

26. Overweight/

27. exp body weight changes/

28. (weight adj1 (loss or lose or losing or lost or maintain* or reduc* or decreas* or chang*)).tw.

29. (body mass index or BMI).tw.

30. waist circumference*.tw.

31. (waist adj4 ratio).tw

32. (body fat or adiposity).tw.

33. or/19-32

34. 11 and 18 and 33

35. exp animals/ not humans/

36. 34 not 35

37. (letter or editorial or comment* or conference).pt.

38. 36 not 37
