# Supplementary material for: Effects of workplace-based dietary and/or physical activity interventions for weight management targeting healthcare professionals: a systematic review of randomised controlled trials
Source: BMC Obes. 2014 Nov 14;1:23. doi: 10.1186/s40608-014-0023-3 (PMC4511014; doi:10.1186/s40608-014-0023-3)
Supplement: Additional file 2: — Reference list of included and excluded studies. [file 40608_2014_23_MOESM2_ESM.docx]

**Additional file 2: Reference lists of included and excluded studies**

**Included studies**

1. Aldana, SG, Greenlaw, RL, Diehl HA, Salberg A, Merrill, RM, Ohmine S: **The effects of**

**a worksite chronic disease prevention program**. *J Occup Environ Med* 2005; **47**: 558-564.

2. Armitage CJ, Conner M: **Efficacy of a minimal intervention to reduce fat intake**. *Soc Sci Med* 2001; **52**: 1517-1524.

3. Barratt A, Reznik R, Irwig L, *et al*.: **Work-site cholesterol screening and dietary**

**intervention: The Staff Healthy Heart Project**. *Am J Public Health* 1994; **84**: 779-782.

4. Brox JI, Frooystein O: **Health-related quality of life and sickness absence in community nursing home employees: Randomized controlled trial of physical exercise**. *Occup Med* 2005; **55**: 558-563.

5. Cockcroft A, Gooch C, Ellinghouse C, Johnston M, Michie S: **Evaluation of a**

**programme of health measurements and advice among hospital staff**. *Occup Med* 1994; **44**: 70-76.

6. Estabrook B, Zapka J, Lemon SC: **Evaluating the implementation of a hospital work-site obesity prevention intervention: Applying the re-aim framework**. *Health Promot Pract* 2012; **13**: 190-197.

6a. Lemon SC, Zapka J, Li W, *et al*.: **Step Ahead. A Worksite Obesity Prevention Trial**

**Among Hospital Employees**. *Am J Prev Med* 2010; **38**: 27-38.

6b. Zapka J, Lemon SC, Estabrook BB, Jolicoeur DG: **Keeping a step ahead: Formative**

**phase of a workplace intervention trial to prevent obesity**. *Obesity* 2007; **15** (Suppl. 1): S27-S36.

7. Gamble RP, Boreham CAG, Stevens AB: **Effects of a 10-week exercise intervention programme on exercise and work capacities in Belfast's ambulancemen**. *Occup Med* 1993; **43**: 85-89.

8. Gerdle, B., Brulin, C., Elert, J., Eliasson, P, Granlund, B: **Effect of a general fitness**

**program on musculoskeletal symptoms, clinical status, physiological capacity, and perceived work environment among home care service personnel**. *J Occup Rehab* 1995*,* **5**: 1-16.

9. Hewitt JA, Whyte GP, Moreton M, Van Someren KA, Levine TS: **The effects of a**

**graduated aerobic exercise programme on cardiovascular disease risk factors in the NHS workplace: A randomised controlled trial**. *J Occup Med Toxicol* 2008; **3**: 7.

10a. Holtermann A, Jorgensen MB, Gram B, *et al*.: **Worksite interventions for preventing**

**physical deterioration among employees in job-groups with high physical work demands: Background, design and conceptual model of FINALE**. *BMC Public Health* 2010; **10**: 120.

10b. Christensen JR, Faber A, Ekner D, Overgaard K, Holtermann A, Sogaard K: **Diet,**

**physical exercise and cognitive behavioral training as a combined workplace based**

**intervention to reduce body weight and increase physical capacity in health care workers – A randomized controlled trial**. *BMC Public Health* 2011; **11**: 671.

10c. Christensen JR, Overgaard K, Carneiro IG, Holtermann A, Sogaard K: **Weight loss**

**among female health care workers-a 1-year workplace based randomized controlled trial in the FINALE-health study**. *BMC Public Health* 2012; **12**: 625.

11. Racette SB, Deusinger SS, Inman CL, *et al*.: **Worksite Opportunities for Wellness**

**(WOW): Effects on cardiovascular disease risk factors after 1 year**. *Prev Med* 2009; **49**: 108-114.

12. Strijk JE, Proper KI, Van Der Beek AJ, Van Mechelen W: **The vital@work study. The**

**systematic development of a lifestyle intervention to improve older workers' vitality and the design of a randomised controlled trial evaluating this intervention**. *BMC Public Health* 2009; **9**: 408.

12a. Strijk JE, Proper KI, Van der Beek AJ, van Mechelen W: **A worksite vitality intervention to improve older workers' lifestyle and vitality-related outcomes: Results of a randomized controlled trial**. *J Epidemiol Community Health* 2012; **66**: 1071-1078.

12b. Strijk JE, Proper KI, van der Beek AJ, *et al*.: **A process evaluation of a worksite vitality intervention among ageing hospital workers**. *Int J Behav Nutr Phys Act* 2011; **8**: 58.

13. von Thiele Schwarz U, Lindfors P, Lundberg U: **Health-related effects of worksite**

**interventions involving physical exercise and reduced workhours**. *Scand J Work Environ Health* 2008; **34**: 179-188.

**Excluded studies**

1. Aittasalo M, Miilunpalo S, Suni J: **The effectiveness of physical activity counseling in a work-site setting. A randomized, controlled trial**. *Patient Education & Counseling* 2004; **55**:193-202.

2. Berendsen BAJ, Hendriks MRC, Verhagen EALM, Schaper NC, Kremers SPJ,

Savelberg HHCM: **Effectiveness and cost-effectiveness of 'BeweegKuur', a combined lifestyle intervention in the Netherlands: rationale, design and methods of a randomized controlled trial**. [*BMC Public Health*](http://www.refworks.com/refworks2/default.aspx?r=references|MainLayout::init) 2011; **11**: 815.

3. Brand R, Schlicht W, Grossman K, Duhnsen R: **Effects of a physical exercise intervention on employees' perceptions quality of life: a randomized controlled trial**. *Soz.Praventiv Med* 2006; **51**: 14-23.

4. Eriksson KM, Westborg CJ, Eliasson MC: **A randomized trial of lifestyle intervention in primary healthcare for the modification of cardiovascular risk factors**. [*Scand.J.Public Health*](http://www.refworks.com/refworks2/default.aspx?r=references|MainLayout::init) 2006; **34**: 453-461.

5. Gilson ND, Puig-Ribera A, McKenna J, Brown WJ, Burton NW, Cooke CB: **Do walking strategies to increase physical activity reduce reported sitting in workplaces: a randomized control trial.** *Int J Behav Nutr Phys Act* 2009; **6**: 43.

6. Glanz K, Patterson RE, Kristal AR Glanz *et al*.: **Impact of work site health promotion on stages of dietary change: the Working Well Trial.** *Health Educ Behav* 1998; **25**: 448-463.

7. Glasgow RE, Terborg JR, Hollis JF, Severson HH, Boles SM: **Take heart: Results from the initial phase of a work-site wellness program**. [*Am.J.Public Health*](http://www.refworks.com/refworks2/default.aspx?r=references|MainLayout::init) 1995; **85**: 209-216.

8. Gomel M, Oldenburg B, Simpson JM, Owen N: **Work-site cardiovascular risk reduction: a randomized trial of health risk assessment, education, counseling, and incentives**. *Am J Public Health* 1993; **83**: 1231–1238.

9. Gorton D, Carter J, Cvjetan B, Ni Mhurchu C: **Healthier vending machines in workplaces: both possible and effective**. *N.Z.Med.J* 2010; **123**: 43-52.

10. Heath GW, Broadhurst CB: **Effects of exercise training and Dietary Behavior Modification on Weight Reduction and Lipoprotein Lipids in Female Hospital Employees**. *Health Values* 1984; **8**: 3-9.

11. Lin JL, Chang SC, Huang SF, Lee MH: **The effect of group aerobic exercise on the health-fitness of female hospital staffs**. [*J Nurs Healthc R*](http://www.refworks.com/refworks2/default.aspx?r=references|MainLayout::init)*es* 2009; **5**: 3-10.

12. McEachan RR, Lawton RJ, Jackson C, Conner M, Meads DM, West RM. **Testing a workplace physical activity intervention: A cluster randomized controlled trial**. *Int J Behav Nutr Phys Act* 2011; **8**: 29

13. Oldervoll L, Ro M, Zwart J-A, Svebak S: **Comparison of two physical exercise programs for the early intervention of pain in the neck, shoulders and lower back in female hospital staff**. *J Rehabil Med* 2001; **33**:156-61.

14. Plotnikoff RC, McCargar LJ, Wilson PM, Loucaides CA: **Efficacy of an e-mail intervention for the promotion of physical activity and nutrition behavior in the workplace context**. [*American Journal of Health Promotion*](http://www.refworks.com/refworks2/default.aspx?r=references|MainLayout::init), 2005; **19**: 422-429.

15. Pohjonen T, Ranta R: **Effects of worksite physical exercise intervention on physical fitness, perceived health status, and work ability among home care workers: five-year follow-up**. [*Prev.Med.*](http://www.refworks.com/refworks2/default.aspx?r=references|MainLayout::init), 2001; **32**: 465-475.

16. Rogers LQ, Gutin B, Humphries MC, *et al*.: **A physician fitness program: enhancing the physician as an "exercise" role model for patients**. [*Teach.Learn.Med*.](http://www.refworks.com/refworks2/default.aspx?r=references|MainLayout::init), 2005; **17**: 27-35.

17. Sorensen G, Thompson B, Glanz K, *et al*.: **Work site-based cancer prevention: Primary results from the working well trial**. [*Am.J.Public Health*](http://www.refworks.com/refworks2/default.aspx?r=references|MainLayout::init), 1996; **86**: 939-947.

18. Tucker SJ, Lanningham-Foster LM, Murphy JN, *et al*.: **Effects of a worksite physical activity intervention for hospital nurses who are working mothers**. *AAOHN* 2011; **59**: 377-386.

19. Tveito TH, Eriksen HR: **Integrated health programme: a workplace randomized controlled trial**. *J Adv Nurs* 2009; **65**: 110–119.

20. Verweij LM, Proper KI, Weel AN, Hulshof CT, van Mechelen W: **Design of the Balance@Work project: systematic development, evaluation and implementation of an occupational health guideline aimed at the prevention of weight gain among employees**. [*BMC Public Health*](http://www.refworks.com/refworks2/default.aspx?r=references|MainLayout::init) 2009; **14**: 461.

21. Zavanela PM, Crewther BT, Lodo L, Florindo AA, Miyabara EH, Aoki MS: **Health and Fitness Benefits of a Resistance Training Intervention Performed in the Workplace**. [*J Strength Condition Res*](http://www.refworks.com/refworks2/default.aspx?r=references|MainLayout::init) 2012; 26: 811-817.
