# Supplementary material for: Effects of workplace-based dietary and/or physical activity interventions for weight management targeting healthcare professionals: a systematic review of randomised controlled trials
Source: BMC Obes. 2014 Nov 14;1:23. doi: 10.1186/s40608-014-0023-3 (PMC4511014; doi:10.1186/s40608-014-0023-3)
Supplement: Additional file 3: — Characteristics of included interventions (stratified by behavioural target). [file 40608_2014_23_MOESM3_ESM.docx]

**Additional file 3: Characteristics of included interventions (stratified by behavioural target)**

| **Study**  **(Year)**  **(Country)**  **(Funding source)** | **Behavioural Target**  **Primary aim *** | **Study design**  **Quality assessment** | **Participants**  **(Intervention: Control)** | **Intervention and control** | | | | | | **Follow-up from baseline** | | **1^o^ outcomes relevant to this systematic review** |
| --- | --- | --- | --- | --- | --- | --- | --- | --- | --- | --- | --- | --- |
|  |  |  |  | **Conditions** | **Components** | | | **Duration** | |  |  |  |
|  |  |  |  |  |  | | |  | |  |  |  |
| Armitage  2001  United Kingdom  Not reported | Diet  Reduce dietary fat intake | RCT:  Individual  Moderate | Mixed  healthcare workforce  Baseline-  412 : 388  follow-up T 1 -  272 : 245 | I: Personalised feedback  On dietary intake (focus on fat) and morbidity and mortality from diet-related conditions  C: General information only | I: a, c, e  C: i | | | 1 day | | T1: 5 months | | Total Fat intake  (mean g/day)  Fat intake  (%)  SF intake (mean g/day) |
| Barratt  1994  Australia  Public and  Private | Diet  Reduce cholesterol levels | RCT: Individual  Weak | Mixed  healthcare workforce  Baseline Ia:Ib:C  310: 144: 259  Follow Up  T1- 417 (total)  T2 -430 (total) | Ia: Screening, Workbook + quizzes + shopping guidelines + recipes, 3-minute, video, monitoring sheet of advised dietary behaviour changes  Ib: Screening, Nutrition course 5 x 1 hour session concerning fibre, fat and dietary  change + workbook + tasting recipes  C: Screening and brief advice | 1a: a,c, e  Ib: a, c, e  C:c | | | 5 weeks | | T1: 3 months  T2: 6 months | | Energy intake (MJ)  Total fat intake (%)  SF intake (% total energy)  Fibre (g/MJ)  Body weight (Kg) |
| Von Thiel  Schwarz  2008  Sweden  Public and Private | Physical activity  Improve health and well-being | RCT: Cluster  Randomised by healthcare organisation  Moderate | Female Dentists  Baseline-  62: 65  follow-up T1- 58:64 follow-up T2- 58: 64 | I: 2.5 hours of weekly mandatory self-selected physical exercise of middle-high intensity, self-monitoring diary.  C: No intervention | I: a, f  C: J | | | 12 months | | T1: 6 months  T2: 12 months | | Physical activity levels  Waist-to-hip ratio |
| **Study**  **(Year)**  **(Country)**  **(Funding source)** | **Behavioural Target**  **Primary aim *** | **Study design**  **Quality assessment** | **Participants**  **(Intervention: Control)** | **Intervention and control** | | | | | | **Follow-up from baseline** | | **1^o^ outcomes relevant to this systematic review** |
|  |  |  |  | **Conditions** | **Components** | | | | **Duration** |  |  |  |
|  |  |  |  |  |  | | | |  |  |  |  |
| Hewitt  2008  United Kingdom  Not reported | Physical activity  Improve cardiovascular health | RCT: Individual  Strong | Cytology staff  Baseline- 12:8  follow-up T 1- 12:8  follow-up T 2- 12:8  follow-up T 3- 12:8 | I: Week 1-8  Individualised progressive exercise brisk walking/light jogging, 4 times/week  Week 1-4: Intensity: 65 % VO_2_peak;Duration: 22.5 min +2.5 min. wk^-1^  Weeks 4-8: Intensity: 65 % VO_2_ peak + 2.5 bpm.wk^-1^; Duration: 30 min  Week 8-12:Individual maintenance of programme  C: Instructed to maintain their current PAL offered intervention at end of the trial | I: h  C:J | | | | 12 weeks | T1: 4 weeks  T2: 8 weeks  T3: 12 weeks | | Body weight  BMI |
| Brox  2005  Norway  Not reported | Physical activity  Reduce sick leave | RCT: Individual  Weak | Nursing home employees  Baseline-63:56  follow-up T 1- 39:42 | I: Light exercise lasting for 1 hour based on an aerobic fitness model Storheim K, Bø K. Classes regarding physical exercise, nutrition and stress management offered. C: No intervention | I: h  C:J | | | | 6 months | T 1: 6 months | | Leisure-time  physical activity |
| Gerdle  1995  Sweden  Public | Physical activity  Improve fitness | RCT: Individual  Weak | Female Homecare Personnel  Baseline 46:49  follow-up T 1-32:45 | I: Training 1 hour twice/week promoting physical fitness: Warming up movements, exercises of coordination, general strength exercises, aerobic profiled activities with 2 or 3 intervals aiming at a stretching of the muscle groups. All activities performed to music. C: No intervention. Intervention repeated for control group after 1 year. | I: h  C: j | | | | 12 months | T1: 12 months | | Body weight (Kg) |
| **Study**  **(Year)**  **(Country)**  **(Funding source)** | **Behavioural Target**  **Primary aim *** | **Study design**  **Quality assessment** | **Participants**  **(Intervention:**  **Control)** | **Intervention and control** | | | | | | **Follow-up from baseline** | | **1^o^ outcomes relevant to this systematic review** |
|  |  |  |  | **Conditions** | | | **Components** | | **Duration** |  |  |  |
|  |  |  |  |  | | |  | |  |  |  |  |
| Gamble  1993  United Kingdom  Not reported | Physical activity  Improve physical fitness | RCT: Individual  Moderate | Paramedics  Baseline- 8:6  follow-up T 1 -8:6 | I: 2 sessions per week  1st session- Flexibility exercises  Continuous indoor football  2nd session- warm-up, flexibility session alternating aerobic and strength exercises sprinting and bounding exercises from week 5  Heart rates were monitored to ensure training workloads were achieved.  C:No intervention | | | I: h  C:J | | 10 weeks | T1: 11 weeks | | Body weight (Kg)  Body fat % |
| Christensen  2011  Denmark  Public | Diet and Physical activity  Weight loss | RCT: Cluster  Randomised by daily contact at work between participants  Strong | Mixed healthcare workforce  Baseline 54: 44  follow-up T 1 - 54:44  follow-up T 2- 54:44 | I: Diet  Individual dietary plan with energy deficit of 1200 Kcal/day  I Physical activity:  Strengthening exercises, Leisure time aerobic exercise ,Log books  I other: Cognitive Behavioural Training, weight check C: Monthly oral presentations on diet | | | I Diet: a, c  I physical activity: c, h  I other: a  C:i | | 12 months | T 1: 3 months  T 2: 12 months | | Body weight (Kg)  BMI  Waist circumference  Body fat %  Waist-hip ratio |
| Strijk  2011  Netherlands  Public | Diet and Physical activity  Improve health and well-being | RCT: Individual  Moderate | Mixed healthcare workforce  Baseline-367:363  follow-up T 1-293:282 | I Diet: Written information  Free fruit at the guided yoga and workout group sessions. Fruit intake diary  I Physical Activity: (Guided group session of yoga- Guided workout group session- aerobic and resistance exercises, unguided aerobic exercising of vigorous activity (e.g. fitness, running, spinning), physical activity diary  C: Written information only. | | | I Diet: a  I Physical activity: a, h  C: i | | 6 months | T1: 6 months | | Fruit intake  PA-sports activities, VPA, MPA |
| **Study**  **(Year)**  **(Country)**  **(Funding source)** | **Behavioural Target**  **Primary aim*** | **Study design**  **Quality assessment** | **Participants**  **(Intervention:**  **Control)** | **Intervention and control** | | | | | | | **Follow-up from baseline** | **1^o^ outcomes relevant to this systematic review** |
|  |  |  |  | **Conditions** | **Components** | | | | **Duration** | |  |  |
|  |  |  |  |  |  | | | |  | |  |  |
| Lemon  2010  USA  Public | Diet and Physical activity  Weight gain prevention | RCT: Cluster  Randomised by medical centre  Moderate | Mixed healthcare workforce  Baseline-386:420  follow-up T 1-344:387  follow-up T 2 299:349 | I Diet: Cafeteria signs and events, healthy menu options, weekly seasonal farmers’ market, recipe books, Brochures, booklets, flyers on healthy eating, monitoring kits  I: Physical activity: Cafeteria events; Stairway signs, Walking Groups, Health and Fitness Day, monthly 30-minute workshop on strength training, Brochures, booklets, flyers, monitoring kits, group and individual prizes  I Other: Interpersonal support, group challenges  C: No intervention | I Diet: a, b, e  I Physical: a, d, g, h  C:j | | | | 12 months | | T 1: 12 months  T 2: 24 months | BMI |
| Racette  2009  USA  Public | Diet and Physical activity  Weight control | RCT: Cluster  Randomised by medical centre  Weak | Mixed Healthcare Workforce  Baseline-84:67  follow-up T 1- 68:55  follow-up T 2- 68:55 | I Diet: Healthy snack cart, weightwatchers group meeting, individualized feedback, rewards.  I Physical activity: Pedometers, walking maps, on-site group exercise programme, individualized feedback, rewards.  I Other: Kick-off event, Monthly lunchtime seminars, Monthly newsletters, Team competitions, Participation cards  C: Assessment only | I Diet: a,, c, d, e  I Physical activity: a, c, d, h  I Other: a, d, i  C: i | | | | 12 months | | T 1: 6 months  T 2: 12 months | Fruit and vegetable intake servings/day  Physical activity patterns  Body weight (Kg), BMI (Kg/m^2^), Waist circumference (cm), Body fat % |
| **Study**  **(Year)**  **(Country)**  **(Funding source)** | **Behavioural Target**  **Primary aim*** | **Study design**  **Quality assessment** | **Participants**  **(Intervention:**  **Control)** | **Intervention and control** | | | | | | | **Follow-up from baseline** | **1^o^ outcomes relevant to this systematic review** |
|  |  |  |  | **Conditions** | | **Components** | | | **Duration** | |  |  |
|  |  |  |  |  | |  | | |  | |  |  |
| Aldana  2005  USA  Not reported | Diet and Physical activity  Promote knowledge of healthy behaviours | RCT: Individual  Moderate | Mixed healthcare workforce  Baseline-66:79  follow-up T 1-64:79  follow-up T 2-64:79 | I Diet: Lectures on diet, shopping tours and cooking demonstrations, preset dietary goals, workbook and assignments  I Physical activity: Lectures on exercise, preset exercise goals – progressive work to min 30 mins exercise/day, pedometer, exercise log, workbook assignments  I Other: Lectures, workbook and assignments  C: Waiting list | | I Diet: a, e  I Physical activity: a, h  I Other: a  C:j | | | 4 weeks | | T1 : 6 Weeks  T2: 6 months | Total energy intake  Total dietary fat, SF  % energy intake from fat,  Fibre intake from fruit and vegetables  Fruit ,vegetable, servings  Total steps/week  Body weight (Kg)  BMI, Body fat % |
| Cockroft  1994  United Kingdom  Not reported | Diet and Physical activity  Promote healthy living | RCT: Individual  Weak | Mixed Healthcare workforce  Baseline-144:153  follow-up T 1-40:43 | I Diet and Physical activity: Baseline measurements reviewed, advice provided, individual 6month target 'contract’, leaflets. Targets included weight loss, a certain frequency of exercise, giving up smoking, or cutting down fatty foods to a certain level.  C: Copy of measurements only | | I Diet and Physical activity: a, c, e  I Control: i | | | 6 months | | T 1: 6 months | Diet Score  Free-time exercise  BMI |

*** As reported in primary paper**

**Abbreviations: N= number, I=intervention, C= control a=behavioural intervention, b=environmental intervention, c=health check (s), d=incentive (s), e=active and continuous promotion of healthy choices, f=working practices and policies, g=supportive physical environment, h=recreational opportunities, i=informational, j=no intervention, T= time, SF = saturated fat, PUFA=polyunsaturated fatty acids, MUFA=monounsaturated fatty acid intake, PA=Physical activity, PAL = Physical activity level, VPA, Vigorous physical activity, MVPA= moderate vigorous physical activity**
