# Supplementary material for: Effects of workplace-based dietary and/or physical activity interventions for weight management targeting healthcare professionals: a systematic review of randomised controlled trials
Source: BMC Obes. 2014 Nov 14;1:23. doi: 10.1186/s40608-014-0023-3 (PMC4511014; doi:10.1186/s40608-014-0023-3)
Supplement: Additional file 4: — Quality assessment of included randomised controlled trials (structured by year of publication). [file 40608_2014_23_MOESM4_ESM.docx]

**Additional file 4: Quality assessment of included randomised controlled trials (structured by year of publication)**

| **INTERVENTION** | **SELECTION**  **BIAS** | **STUDY**  **DESIGN** | **CONFOUNDERS** | **BLINDING** | **DATA COLLECTION METHODS** | **WITHDRALWS AND DROP OUTS** | **GLOBAL**  **RATING** |
| --- | --- | --- | --- | --- | --- | --- | --- |
| Christensen 2011 | Very likely  60 - 79% Agreement  MODERATE | RCT  Yes Yes Yes  STRONG | No  N/A  STRONG | No  Can’t tell  MODERATE | Yes  Yes  STRONG | Yes  80-100%  STRONG | STRONG |
| Strijk 2011 | Very likely  Less than  60% Agreement  WEAK | RCT  Yes Yes Yes  STRONG | No  N/A  STRONG | Can’t tell  Can’t tell  MODERATE | Yes  Yes  STRONG | Yes  60 - 79%  MODERATE | MODERATE |
| Lemon 2010 | Very likely  Less than  60% Agreement  WEAK | RCT  Yes Yes Yes  STRONG | Yes  80 – 100%  STRONG | Can’t tell  Can’t tell  MODERATE | Yes  Yes  STRONG | Yes  60 - 79%  MODERATE | MODERATE |
| Racette 2009 | Not likely  Less than  60% Agreement  WEAK | CCT  Yes No N/A  STRONG | Yes  Can’t tell  WEAK | Can’t tell  Can’t tell  MODERATRE | Yes  Yes  STRONG | Yes  80 -100%  STRONG | WEAK |
| **INTERVENTION** | **SELECTION**  **BIAS** | **STUDY**  **DESIGN** | **CONFOUNDERS** | **BLINDING** | **DATA COLLECTION METHODS** | **WITHDRALWS AND DROP OUTS** | **GLOBAL**  **RATING** |
| Von Thiele 2008 | Somewhat  likely  80-100% Agreement  MODERATE | CCT  Yes No N/A  STRONG | No  N/A  STRONG | Can’t tell  Yes  MODERATE | Can’t tell  Can’t tell  WEAK | Yes  80-100%  STRONG | MODERATE |
| Hewitt 2008 | Somewhat  likely  Can’t tell  MODERATE | CCT  Yes Yes No  STRONG | No  N/A  STRONG | Can’t tell  Can’t tell  MODERATE | Yes  Yes  STRONG | Yes  80-100%  STRONG | STRONG |
| Aldana 2005 | Very likely  80-100% Agreement  STRONG | RCT  Yes Yes Yes  STRONG | No n/a  STRONG | Yes  Can’t tell  WEAK | Yes  Yes  STRONG | Yes  80-100%  STRONG | MODERATE |
| Brox 2005 | Not likely  Less than  60% Agreement  WEAK | CCT  Yes No N/A  STRONG | No  n/a  STRONG | No  Can’t tell  MODERATE | Can’t tell  Can’t tell  WEAK | No  60 - 79%  MODERATE | WEAK |
| **INTERVENTION** | **SELECTION**  **BIAS** | **STUDY**  **DESIGN** | **CONFOUNDERS** | **BLINDING** | **DATA COLLECTION METHODS** | **WITHDRALWS AND DROP OUTS** | **GLOBAL**  **RATING** |
| Armitage 2001 | Somewhat likely  Can’t tell  MODERATE | CCT  Yes No N/A  STRONG | Yes  80-100%  STRONG | Can’t tell  Can’t tell  MODERATE | Yes  Yes  STRONG | No  60 - 79%  MODERATE | MODERATE |
| Gerdle 1995 | Somewhat  likely  80-100% Agreement  MODERATE | CCT  Yes No N/A  STRONG | Yes  Can’t tell  WEAK | Can’t tell  Can’t tell  MODERATE | Can’t tell  Can’t tell  WEAK | Yes  60 - 79%  MODERATE | WEAK |
| Barratt 1994 | Somewhat likely  60 - 79% Agreement  MODERATE | CCT  Yes No N/a  STRONG | Can’t tell  N/A  WEAK | Can’t tell  Can’t tell  MODERATE | Can’t tell  Can’t tell  WEAK | No  60 - 79%  MODERATE | WEAK |
| **INTERVENTION** | **SELECTION**  **BIAS** | **STUDY**  **DESIGN** | **CONFOUNDERS** | **BLINDING** | **DATA COLLECTION METHODS** | **WITHDRALWS AND DROP OUTS** | **GLOBAL**  **RATING** |
| Cockroft 1994 | Not likely  Less than  60%  agreement  WEAK | CCT  (Individual)  Yes No N/A  STRONG | Can’t tell  Can’t tell  WEAK | Can’t tell  Can’t tell  MODERATE | Yes  No  MODERATE | Yes  60 - 79%  MODERATE | WEAK |
| Gamble 1993 | Not likely  80-100% Agreement  WEAK | CCT  Yes No N/A  STRONG | No  N/A  STRONG | Can’t tell  Can’t tell  MODERATE | Yes  Yes  STRONG | N/A  80-100%  STRONG | MODERATE |

**Abbreviations: RCT = Randomised controlled trial; CCT = Controlled clinical trial;**

**N/A = Not applicable**
